# Long‐term cognitive outcomes in tuberous sclerosis complex

**DOI:** 10.1111/dmcn.14356

**Published:** 2019-09-19

**Authors:** Charlotte Tye, Fiona S Mcewen, Holan Liang, Lisa Underwood, Emma Woodhouse, Edward D Barker, Fintan Sheerin, John R W Yates, Patrick F Bolton, Tuberous Sclerosis 2000 Study Group, N Higgins, V Attard, A Clarke, FV Elmslie, AK Saggar, D Baines, BA Kerr, C Brayne, I Carcani‐Rathwell, C Connolly, M Clifford, A Lydon, F Oluwo, H Rogers, C Srivastava, J Steenbruggen, JA Cook, C Falconer, DM Davies, JR Sampson, AE Fryer, M Haslop, Y Granader, PD Griffiths, A Hunt, WWK Lam, JC Kingswood, ZH Miedzybrodzka, H Crawford, PJ Morrison, FJK O'Callaghan, SG Philip, S Seri, R Sheehan‐Dare, CH Shepherd

**Affiliations:** ^1^ Department of Child & Adolescent Psychiatry Institute of Psychiatry, Psychology & Neuroscience King's College London London UK; ^2^ Social Genetic & Developmental Psychiatry Centre Institute of Psychiatry, Psychology & Neuroscience King's College London London UK; ^3^ Department of Biological and Experimental Psychology School of Biological and Chemical Sciences Queen Mary University of London London UK; ^4^ Great Ormond Street Hospital NHS Trust London UK; ^5^ Institute of Child Health University College London London UK; ^6^ Department of Population Health University of Auckland Auckland New Zealand; ^7^ Forensic and Neurodevelopmental Sciences Institute of Psychiatry, Psychology & Neuroscience King's College London London UK; ^8^ South London and Maudsley NHS Trust London UK; ^9^ Department of Psychology Institute of Psychiatry, Psychology & Neuroscience King's College London London UK; ^10^ Department of Neuroradiology Oxford University Hospital NHS Foundation Trust Oxford UK; ^11^ Department of Medical Genetics Cambridge University Cambridge UK

## Abstract

**Aim:**

To investigate the interdependence between risk factors associated with long‐term intellectual development in individuals with tuberous sclerosis complex (TSC).

**Method:**

The Tuberous Sclerosis 2000 Study is a prospective longitudinal study of individuals with TSC. In phase 1 of the study, baseline measures of intellectual ability, epilepsy, cortical tuber load, and mutation were obtained for 125 children (63 females, 62 males; median age=39mo). In phase 2, at an average of 8 years later, intellectual abilities were estimated for 88 participants with TSC and 35 unaffected siblings. Structural equation modelling was used to determine the risk pathways from genetic mutation through to IQ at phase 2.

**Results:**

Intellectual disability was present in 57% of individuals with TSC. Individuals without intellectual disability had significantly lower mean IQ compared to unaffected siblings, supporting specific genetic factors associated with intellectual impairment. Individuals with TSC who had a slower gain in IQ from infancy to middle childhood were younger at seizure onset and had increased infant seizure severity. Structural equation modelling indicated indirect pathways from genetic mutation, to tuber count, to seizure severity in infancy, through to IQ in middle childhood and adolescence.

**Interpretation:**

Early‐onset and severe epilepsy in the first 2 years of life are associated with increased risk of long‐term intellectual disability in individuals with TSC, emphasizing the importance of early and effective treatment or prevention of epilepsy.

**What this paper adds:**

- Intellectual disability was present in 57% of individuals with tuberous sclerosis complex (TSC).
- Those with TSC without intellectual disability had significantly lower mean IQ compared to unaffected siblings.
- Earlier onset and greater severity of seizures in the first 2 years were observed in individuals with a slower gain in intellectual ability.
- Risk pathways through seizures in the first 2 years predict long‐term cognitive outcomes in individuals with TSC.

## What this paper adds

- Intellectual disability was present in 57% of individuals with tuberous sclerosis complex (TSC).
- Those with TSC without intellectual disability had significantly lower mean IQ compared to unaffected siblings.
- Earlier onset and greater severity of seizures in the first 2 years were observed in individuals with a slower gain in intellectual ability.
- Risk pathways through seizures in the first 2 years predict long‐term cognitive outcomes in individuals with TSC.

This article is commented on by Curatolo on page https://doi.org/10.1111/dmcn.14371 of this issue.

**Abbreviation**
TSC: Tuberous sclerosis complex

Tuberous sclerosis complex (TSC) is a genetic disorder caused by a mutation in the *TSC1* or *TSC2* genes. The TSC proteins hamartin and tuberin form a heterodimeric complex involved in intracellular signalling and the activation of mammalian target of rapamycin complex 1, which plays an important role in the regulation of cell proliferation and differentiation.^1^ In addition to the primary mutation event, stochastic second‐hit mutations in the normal copy allele during embryogenesis lead to marked upregulation of the mammalian target of rapamycin pathway and consequently the development of hamartomatous lesions and random variation in the size and location of lesions, leading to highly variable phenotypic expression. In the brain, subependymal nodules in the ventricular walls and cortical tubers at the grey–white matter interface arise and are associated with a high risk of epilepsy, which occurs in up to 90% of individuals and typically begins in the first year of life. Epileptic (infantile) spasms are common, but are often preceded by or coexist with focal seizures.^2^ More than half of patients with TSC have some form of intellectual impairment and approximately one‐third have profound disability, while a significant proportion does not display impairment.^3^ A bimodal distribution of IQ in individuals with TSC has been reported,^4^ although whether this reflects a genotype–phenotype correlation or the effect of epilepsy is unclear.^5^ With the advent of mammalian target of rapamycin inhibitory treatment for several complications of TSC and speculation that it may hold promise for improving cognitive development, it has become especially important to characterize the longitudinal pathways leading to intellectual impairment in individuals with TSC, to identify the risk processes that could be targets for treatment.

Intellectual development in individuals with TSC is likely the result of a complex interplay of the wide range of identified risk factors. Several previous studies have relied on univariate analyses of single predictors at single time points. On a genetic level, *TSC2* mutations are associated with a more severe phenotype, including lower intellectual ability.^6^, ^7^ Yet, profound disabilities can be observed in individuals with a *TSC1* mutation, as well as high IQ in individuals with a *TSC2* mutation. The extent of brain involvement indexed by tuber count and load has also been correlated with lower IQ;^8^, ^9^, ^10^, ^11^ however, a large tuber burden has been reported in individuals with normal IQ.^12^ Early‐onset seizures and epileptic spasms are strongly associated with intellectual disability in individuals with TSC.^12^, ^13^, ^14^ Prospective longitudinal studies indicate poor intellectual development in the first 2 years of life in infants with spasms,^15^ and impaired development after the onset of spasms compared to non‐spasm seizures.^16^

Multifactorial analyses that can test the joint effects of these risk factors have suggested that age at seizure onset is the sole variable that can be used to independently predict intellectual outcome in children and adults.^12^, ^17^ However, these previous analyses have relied on retrospective reports and chart review to characterize epilepsy in clinic‐referred samples. Overcoming some of these limitations, our previous multivariate analysis of IQ collected in the longitudinal, prospective, and nationally ascertained Tuberous Sclerosis 2000 Study cohort indicated an indirect pathway linking the type of genetic mutation to cortical tuber load, to epilepsy severity (latent factor for seizure severity in the first 2 years of life and current seizure severity), through to intellectual ability, at an average age of 39 months.^18^ This suggests a cascading risk pathway from genetic mutation to intellectual outcome in early childhood. The pathway leading to variability in intellectual outcomes in the longer term (beyond early childhood) is not well mapped; this has clinically relevant implications for identifying early predictors of later developmental outcomes.

We aimed to build on previous work by investigating: (1) the distribution of IQ and rates of intellectual disability later in childhood and adolescence in individuals with TSC and unaffected siblings; (2) the effect of genetic mutation as well as brain‐ and epilepsy‐related risk factors on long‐term change in IQ over childhood and adolescence, by grouping individuals according to a faster (increase in IQ) versus slower (decrease in IQ) gain in skills; and (3) multifactorial longitudinal pathways to individual differences in intellectual ability in individuals with TSC using structural equation modelling. We hypothesized that early seizure severity would mediate the relationship between genetic mutation and long‐term intellectual outcomes in TSC.

## Method

### Participants

The Tuberous Sclerosis 2000 Study is a population‐based, prospective, longitudinal study of the natural history of TSC (for full details, see Yates et al.^2^ and Appendix S1, online supporting information). In phase 1 of the study (2001–2005), 125 participants met the diagnostic criteria for TSC at 0 to 16 years old and completed the study assessments (63 females, 62 males, median age=39mo, range=4–254mo). At the initial assessment, a full medical history was obtained and a physical examination carried out using a standardized protocol. Full details of the study assessment protocol have been reported elsewhere.^2^ During phase 2 of the study (2012–2015), research psychologists gathered information on the intellectual abilities of 88 participants (49 females, 39 males, median age=148mo, range=93–323mo), at an average of 8 years 4 months (5y 6mo–10y 10mo) after phase 1. In addition, to control for potential familial influences on intellectual outcome, 35 unaffected siblings (14 females, 21 males, median age=145mo, range=78–259mo) participated in phase 2 of the study. Nineteen siblings were recruited from seven families (17 full siblings, 1 half‐sibling, and 1 dizygotic twin).

### Measures

#### Genetic testing

A causal mutation was determined for 96 children (*TSC1*,* n*=19; *TSC2*,* n*=77). A pathogenic mutation could not be identified in seven children; genetic testing was not carried out in 22 children.

#### Brain imaging

Copies of clinical brain scans were obtained from the hospitals where imaging had been conducted during phases 1 and 2, whenever possible (*n*=109; Appendix S1). The scans were reviewed and rated without knowledge of other clinical details by Nicholas Higgins (phase 1) and Fintan Sheerin (phase 2) using a prespecified coding system that recorded the number and lobar location of cortical tubers and subependymal nodules. The interrater reliability of this procedure has previously been shown to be acceptable.^19^ Tuber count was summated for each major lobe of the brain (Figs S1 and S2, online supporting information).

#### Epilepsy severity

Using information derived from a specially devised interview schedule, seizure diary, and medical records, epilepsy features were combined to generate a seizure severity score using the Early Childhood Epilepsy Severity Scale.^20^ Exploratory factor analysis was conducted to ascertain the loadings of each Early Childhood Epilepsy Severity Scale feature to severity scores (Fig. S3, online supporting information), with the following parameters loading onto one factor: number of seizure types; time over which seizures occurred (duration); seizure frequency at most severe; number of antiepileptic drugs used; and treatment response. Severity scores were calculated for four time points: the first year of life (*n*=120); the second year of life (*n*=120); the 3‐month period leading up to the phase 1 assessment (*n*=125); and the 3‐month period leading up to the phase 2 assessment (current seizure severity; *n*=94). To disentangle the effect of epileptic spasms from other seizure types (including generalized and focal seizures, defined using International League Against Epilepsy classification) on intellectual outcome, spasm severity was separately coded from ‘non‐spasm seizure severity’ for the first and second year of life, when spasms tend to occur (Appendix S1).

#### Intellectual ability and adaptive behaviour

Following previous work,^12^, ^17^ an estimated IQ was available for 121 participants in phase 1 and 88 in phase 2 (Appendix S1). In phase 1, intellectual abilities were assessed using the Mullen Scales of Early Learning in participants up to 68 months of age^21^ or the Vineland Adaptive Behavior Scales extended survey parental interview.^22^ In phase 2, participants were administered the Wechsler Abbreviated Scale of Intelligence, Second Edition,^23^ British Picture Vocabulary Scale,^24^ and/or the Vineland Adaptive Behavior Scales, Second Edition.^25^

### Statistical analysis

Factor scores resulting from factor analysis of tuber load and seizure severity were used in group analyses and structural equation modelling (Figs S2 and S3).

#### Distribution of IQ in participants with TSC and their unaffected siblings

Group differences in the estimated IQ of individuals with TSC and unaffected siblings were analysed with *t*‐tests in Stata 10 (StataCorp LLC, College Station TX, USA) using a robust cluster command to estimate standard errors, which takes non‐independent observations into account.

#### Factors associated with developmental change in intellectual ability in TSC

Grouping according to developmental change in skills was defined by a significant change in IQ from phase 1 to 2 (+15 IQ points for a faster gain in skills, −15 IQ points for a slower gain in skills). Mann–Whitney *U* tests for non‐normally distributed data were conducted with group (faster vs slower gain in skills) as the between‐participant variable and risk factor (e.g. seizure severity) as the dependent variable. Participants with no significant change in IQ were not included in the group comparisons; descriptive data are provided in Table S1 (online supporting information). *χ* ^2^ tests were used to examine the association between mutation and history of epileptic spasms on change. Spearman's correlations were calculated between change in IQ and ordinal epilepsy parameters. Group differences in estimated IQ (normally distributed) by history of epileptic spasms and status epilepticus were tested by analysis of variance.

#### Longitudinal risk pathways to intellectual outcomes in TSC

Structural equation modelling was conducted using an indirect mediation model in MPlus v7.31 (Muthén & Muthén, Los Angeles, CA, USA). Factor scores for spasm and non‐spasm severity in years 1 and 2 were loaded onto latent variables. Factor loadings for tuber load and seizure severity, and raw scores for estimated IQ in phases 1 and 2 were entered as measured variables. To account for missing values, full information maximum likelihood estimation with robust standard errors was used. The bootstrap model (1000 resamples) was used to estimate the standard errors of the parameter estimates and the bias‐corrected confidence intervals (CIs) of the indirect effects. The estimated indirect pathways were restricted by only considering indirect pathways where there was a significant coefficient for each path. Indirect effects were considered statistically significant if the corresponding 95% CI did not include zero.

### Ethical approval

A medical ethics committee (NRES West Midlands – Edgbaston) approved the study protocol. Written informed consent was obtained from all study participants and/or their caregivers before the assessments began.

## Results

### Distribution of IQ in individuals with TSC and unaffected siblings

**Figure 1:**
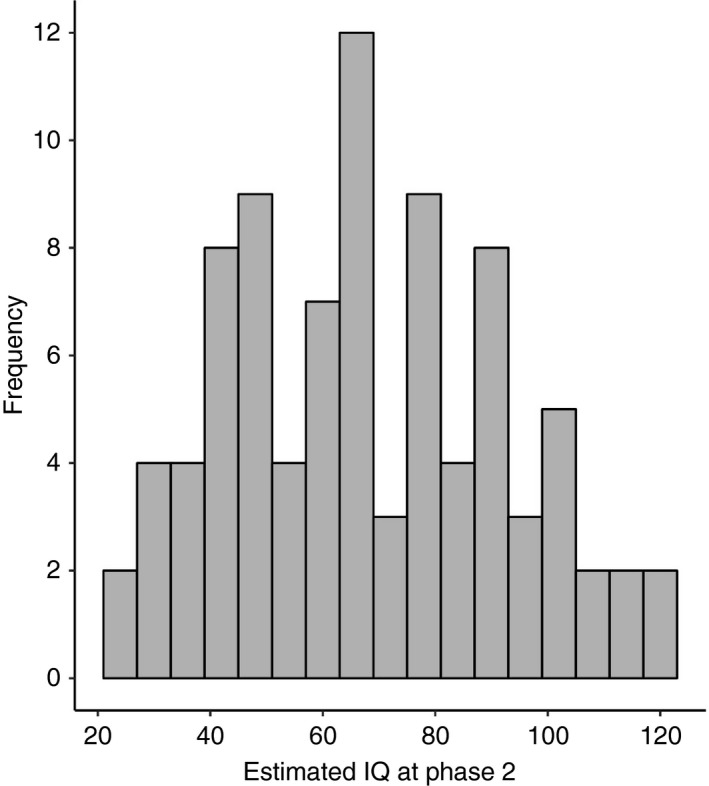
Distribution of intellectual ability in phase 2 of the Tuberous Sclerosis 2000 Study.

The mean estimated IQ at phase 1 in participants with TSC was 67.4 (range 20–132), with 62.4% (*n*=78) under 70 (indicating intellectual disability). The mean estimated IQ at phase 2 in participants with TSC was 68.1 (range 26–119), with 56.8% (*n*=50) under 70 (Fig. 1). No individual had profound disability (estimated IQ ≤20), 11.4% (*n*=10) had severe intellectual disability (20–34), and 45% (*n*=40) had mild‐to‐moderate intellectual disability (35–70).

**Figure 2:**
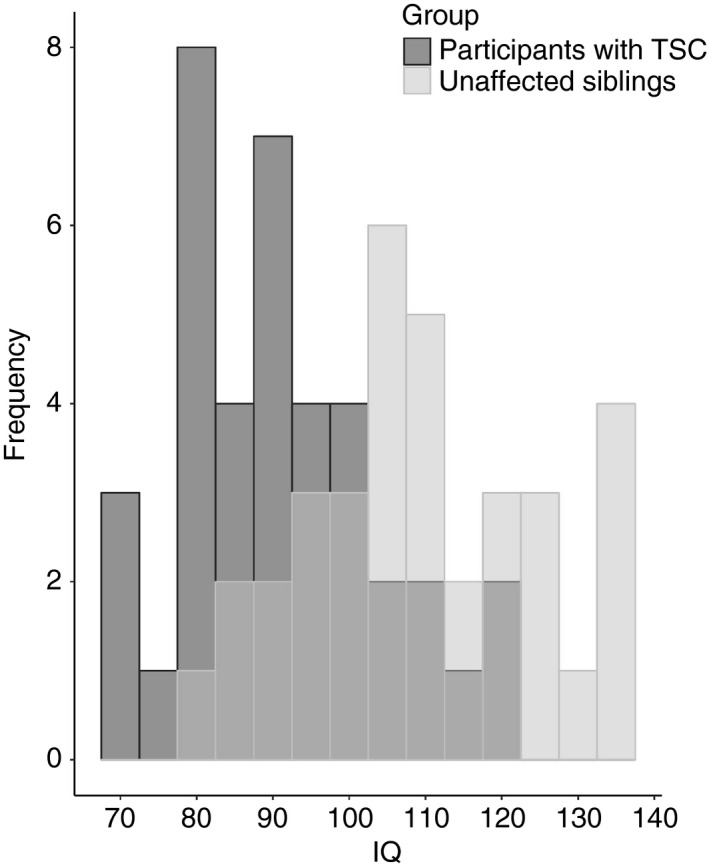
Distribution of intellectual ability in participants with tuberous sclerosis complex (TSC) with an IQ greater than 69 and unaffected siblings.

We compared the IQ scores of individuals with TSC with an IQ in the typical range (>69; *n*=38; 21 females, 17 males, median age=161mo, range=93–323mo), with those of unaffected siblings (*n*=35; 14 females, 21 males; median age=145mo, range=78–259mo; Fig. 2). The mean IQ of unaffected siblings (mean=110.06, SD=15.12) was significantly higher than the TSC population (mean=91.26, SD=13.18, *t*=−5.60, *p*<0.001, *d*=1.33). The difference was retained in individuals with TSC who did not have current seizures (*n*=22; mean=92.00, SD=13.28, *t*=−4.58, *p*<0.001, *d*=1.27) or a history of spasms (*n*=25; mean=93.12, SD=13.67, *t*=−4.62, *p*<0.001, *d*=1.18). There was an association between IQ in unaffected siblings and maternal education level, a proxy for socio‐economic status (*n*=25; *r*=0.66, *p*=0.001), but not in TSC (*n*=28; *r*=0.02, *p*=0.94).

### Factors associated with developmental change in intellectual ability in TSC

Of the 85 individuals with IQ data at both phases 1 and 2, 50 participants (58.8%) had no significant change (<15 points) in IQ, 16 showed a relative decrease (18.8%), and 19 showed a relative increase (22.4%). There was no difference in point change in IQ based on intellectual disability status at phase 1 (no intellectual disability=2.72, intellectual disability=−1.12; *F*[1,82]=0.88, *p*=0.35). A relative decrease in IQ may represent slower gain in intellectual ability during development, rather than loss of skills or developmental plateauing; hereafter, this group is described as showing a slower gain in skills. Individuals with a significant change in IQ (decreased vs increased; *n*=35) were compared on a range of potential risk factors (Table S2). The group with a slower gain in intellectual ability showed a higher likelihood of history of status epilepticus, younger age at seizure onset, and increased severity of non‐spasm seizures in the first 2 years of life, compared to the group with a faster gain in ability. Age at seizure onset (*ρ*=0.38, *p*=0.001), history of epileptic spasms (*F*[1,86]=10.95, *p*=0.001), and history of status epilepticus (*F*[1,86]=14.59, *p*<0.001) were each significantly associated with lower estimated IQ at phase 2. Multivariate linear regression indicated that among these three predictors, age at seizure onset (*β*=0.26, *p*=0.02) and status epilepticus (*β*=−0.25, *p*=0.02) were the most strongly associated with estimated IQ; in their presence, a history of spasms was no longer significantly associated (*β*=−0.13, *p*=0.25). Participants with seizure onset after 12 months of age had higher IQ (75.77) at phase 2 compared to participants with seizure onset under 12 months of age (65.02; *t*[80]=−2.50, *p*=0.02, *d*=0.65).

Using point change in IQ as a dimensional measure, there was a significant correlation with age at seizure onset (*ρ*=−0.33, *p*=0.003), severity scores for non‐spasm seizures in years 1 (*ρ*=0.42, *p*<0.001) and 2 (*ρ*=0.25, *p*=0.02), and a trend approaching significance towards increased tuber load (*ρ*=0.22, *p*=0.05). No other correlations were significant (all *p*>0.05).

### Longitudinal risk pathways to intellectual outcome

**Figure 3:**
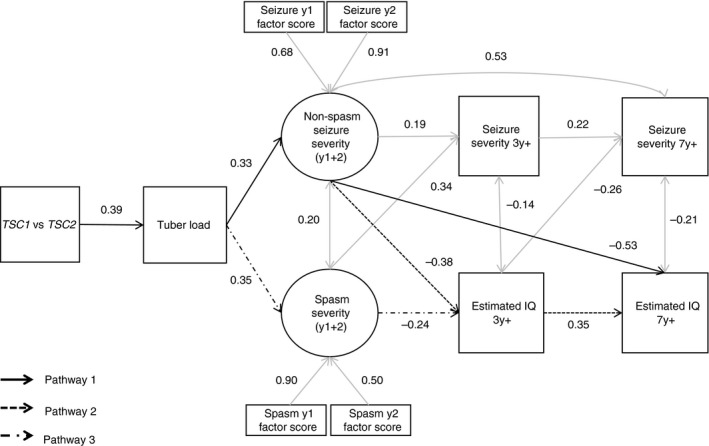
Full mediation model: the paths linking genotype and intellectual outcomes, through tuber load and epilepsy severity, are shown. The ovals represent latent variables, the rectangles observed variables. Only significant paths are shown; absence of a line connecting variables implies no direct effect (path was not significant). The standardized *β* for each path is shown; all paths shown are significant at *p*<0.05.

Bivariate correlations between variables and phase 2 IQ are provided in Tables S2 and S3 (online supporting information). A ‘saturated’ model including all direct and indirect paths linking the constructs of interest was tested. The model yielded a good fit to the data (*χ* ^2^[24]=43.31, *p*=0.009, root mean square error of approximation=0.08 (90% CI=0.04–0.12); standardized root mean square residual=0.06, comparative fit index=0.98). All significant direct paths are shown in Fig. 3.

Three indirect paths were significant: (1) the strongest pathway was indicated through the type of genetic mutation, through tuber load, through non‐spasm seizure severity in the first 2 years of life, and through to IQ at phase 2 (*β*=−4.81, 95% CI=−9.78 to −1.40), explaining 38% of the total indirect effects between genetic mutation and IQ at phase 2 when all pathways were included (72% when restricted to significant pathways only); (2) mutation, through tuber load, through non‐spasm seizure severity, through phase 1 IQ, through to phase 2 IQ (*β*=−1.14, 95% CI=−3.05 to −0.41); and (3) through mutation, through tuber load, through spasm severity, through phase 1 IQ, and through to phase 2 IQ (*β*=−0.72, 95% CI=−3.05 to −0.41). Comparisons of these indirect effects revealed that pathway 1 through non‐spasm seizure severity, through to IQ at phase 2 was significantly larger than pathway 2 through non‐spasm seizure severity, through to IQ at phase 1 (*β*=1.31, 95% CI=0.51–3.35), and pathway 3 through tuber load, through to epileptic spasms and IQ at phase 1 (*β*=0.84, 95% CI=0.31–2.14). Given the effect of age at seizure onset on the trajectories of intellectual development, age at seizure onset was included in a supplementary model; this replicated the same indirect pathways while controlling for the effects of age at onset (Fig. S4 and Appendix S2, online supporting information). To confirm the validity of the estimated IQ, two additional models were executed using: (1) scores derived from the Mullen Scales of Early Learning only at phase 1 and the Wechsler Abbreviated Scale of Intelligence, Second Edition only at phase 2; and (2) Vineland Adaptive Behavior Scales scores only from both phases (Figs S5 and S6, online supporting information). The first model indicated three of the same significant indirect pathways (1, 2, and 4) linking genetic mutation through to IQ at phase 2 described earlier (Appendix S3 and Fig. S5, online supporting information). The second model indicated different distinct pathways linking genetic mutation to adaptive ability at phase 2, with the largest effects shown for a pathway linking type of genetic mutation, through cortical tuber load, through to adaptive ability at phase 2, and epileptic spasms through to adaptive ability at phase 2 (Appendix S4 and Fig. S6, online supporting information). We ran a further model to estimate pathways to phase 1 IQ only (Appendix S5 and Fig. S7, online supporting information), indicating pathways through both non‐spasm and spasm severity; this confirmed our previous findings.^18^

## Discussion

This is the first study to explore long‐term risk pathways to intellectual outcome in individuals with TSC, by examining the interaction between clinical risk factors on the trajectories of intellectual development. The findings demonstrate a robust effect of epilepsy severity in the first 2 years of life, particularly for seizures other than epileptic spasms. This finding holds both when examining change in IQ over an average period of 8 years, and when applying structural equation modelling to explore the indirect longitudinal pathways linking genetic mutation to intellectual ability.

Lower IQ was demonstrated in individuals with TSC compared to unaffected siblings, and IQ scores were truncated in the group with TSC, even in the more able subgroup. Association with maternal education level, a proxy for socio‐economic status, was not indicated in the group with TSC, suggesting lower IQ is not associated with environmental factors and familial influences. This suggests that specific genetic and/or neurological factors are associated with intellectual impairment in individuals with TSC, even in the absence of intellectual disability, and override the effects of socio‐economic status that are seen in typically developing individuals. Intellectual ability in individuals with TSC showed a unimodal distribution in line with findings at a younger age in the same cohort^18^ but in contrast with other findings,^4^ which may reflect a subset of individuals who experience further cognitive decline into adulthood.^26^ A decrease in IQ from early childhood to middle childhood/adolescence, which could reflect either loss of or failure to gain skills over time, was associated with earlier age at seizure onset and increased seizure severity in the first 2 years of life, similar to previous retrospective studies.^12^, ^17^ It is important to conduct follow‐up IQ assessments as cohort members transition to adulthood to track further changes in developmental trajectories.

Importantly, this work extends previous research by mapping the pathways between the type of genetic mutation and IQ across development, while reproducing the risk pathways to short‐term IQ. Lower Wechsler Abbreviated Scale of Intelligence, Second Edition scores were demonstrated in patients with a *TSC2* versus *TSC1* mutation (Table S3), which was not indicated for the Vineland Adaptive Behavior Scales, Second Edition or estimated IQ. This probably reflects the reduced sample size of participants completing the Wechsler Abbreviated Scale of Intelligence, Second Edition and lower frequency of *TSC1* mutations. Sophisticated structural equation modelling indicated a significant indirect pathway linking the type of genetic mutation (*TSC2*), to increased tuber load, to increased severity of seizures in the first 2 years of life, through to intellectual ability in middle childhood and adolescence. This suggests that the association between genetic mutation and long‐term intellectual outcome is mediated by tuber burden and seizure severity in the early years, while controlling for current seizure severity. However, three additional pathways were indicated. First, through early seizure severity and intellectual ability at phase 1 of the study, which supports our previous findings^18^ and suggests seizure severity may operate through childhood intellectual ability; for a subset of individuals, this remains relatively stable. Second, we observed two pathways operating through epileptic spasm severity to intellectual ability at phase 1 through to intellectual ability at phase 2, which suggests that spasms have a direct effect on short‐term intellectual outcomes, in line with previous work.^16^ Yet, the long‐term effects of epileptic spasm treatment on cognitive ability have also been demonstrated.^13^ Therefore, our finding could reflect the evolution of non‐spasm seizures to spasms in the early years, or the effect of additional unmeasured variables. Furthermore, spasm severity may show a greater effect on other developmental outcomes in individuals with TSC, such as adaptive functioning rather than general cognitive ability (supported in Appendix S4 and Fig. S6) and symptoms of autism spectrum disorder. These effects should be systematically explored through repeated assessment of infants with TSC recruited before seizure onset, particularly since it is difficult to discriminate reliably between spasm and non‐spasm seizures.

The demonstration of multiple longitudinal pathways to intellectual outcome may reflect varied and unique developmental trajectories based on risk factors and phenotypic expressions, which may support more specific and targeted therapeutic strategies. Still, the findings converge to support the major contributory role of early seizure severity on outcome; thus, controlling seizures in the first 2 years of life may be key for long‐term intellectual development, as supported by improved short‐term outcomes for infants treated early^13^ or preventatively based on epileptic discharges on electroencephalography.^27^ Recent findings support the predictive validity of certain electroencephalography biomarkers on subsequent seizure onset in infants with TSC,^28^ which demonstrates the potential value of targeting treatments before the onset of epilepsy. Results from early treatment trials will also provide insight into the causal nature of the identified risk pathways that are mediated by early seizure severity.

Several limitations should be taken into account. First, the majority of brain imaging data were derived from routine clinical investigations undertaken at different points during development using different procedures. While tuber load was a key determinant of seizures during the early years, consistent with previous research,^12^ separating epilepsy‐related intellectual disability from that as a consequence of tubers is a challenge, since tubers may not only cause intellectual disability but also hold epileptogenic capability. It is important to conduct in‐depth neurological assessments to determine whether the risk incurred by seizure severity is acting as a marker for unmeasured brain abnormalities, such as altered connectivity between brain regions that cannot be observed on conventional magnetic resonance imaging.^29^ Second, there was no direct pathway linking the type of genetic mutation to epilepsy severity or intellectual outcome. Although not inconsistent with previous studies, it is important to conduct further genetic analyses since certain types of mutations within *TSC1* and *TSC2* can result in varying disease severity.^5^ Third, different measures were used to measure intellectual ability because of a wide age and ability range, and follow‐up assessments were conducted at different intervals. Nevertheless, associations in the predicted direction across analyses have been shown, suggesting robust associations regardless of measurement error. This warrants replication within larger, prospective, longitudinal studies with systematic assessments throughout development.

This study demonstrates a cascading longitudinal pathway from gene to tuber load to early epilepsy severity through to long‐term intellectual ability. These findings warrant the early detection and control of seizures as well as regular neurocognitive assessments and support to improve the intellectual outcomes of individuals with TSC and other early‐onset epilepsy syndromes.

## Supporting information


**Appendix S1:** Methods.

**Appendix S2:** Structural equation modelling with age at seizure onset.

**Appendix S3:** Structural equation modelling for Mullen Scales of Early Learning at phase 1 and Wechsler Abbreviated Scale of Intelligence, Second Edition at phase 2.

**Appendix S4:** Structural equation modelling for the Vineland Adaptive Behaviour Scale at phases 1 and 2.

**Appendix S5:** Structural equation modelling for phase 1 estimated IQ only.

**Figure S1:** Distribution of total tuber count across the cohort.

**Figure S2:** Factor analysis on tuber load in each lobe.

**Figure S3:** Factor analyses on Early Childhood Epilepsy Severity Scale items at each time point.

**Figure S4:** Full mediation model: paths linking genotype and intellectual outcomes with age at seizure onset included.

**Figure S5:** Full mediation model: paths linking genotype and intellectual outcomes using Mullen Scales of Early Learning at phase 1 and Wechsler Abbreviated Scale of Intelligence, Second Edition at phase 2.

**Figure S6:** Full mediation model: paths linking genotype and intellectual outcomes using the Vineland Adaptive Behaviour Scale at phases 1 and 2.

**Figure S7:** Full mediation model: paths linking genotype and intellectual ability at phase 1 only.

**Table S1:** Clinical characteristics of sample subgroups with: (1) no change, (2) a relative increase, or (3) a relative decrease in IQ from phase 1 to phase 2

**Table S2:** Spearman correlations between main phenotypic features

**Table S3:** Group differences by genotype
