# Supplementary material for: Long‐term cognitive outcomes in tuberous sclerosis complex
Source: Dev Med Child Neurol. 2019 Sep 19;62(3):322–9. doi: 10.1111/dmcn.14356 (PMC7027810; doi:10.1111/dmcn.14356)
Supplement: Supplementary file 1 — Appendix S1: Methods. [file DMCN-62-322-s001.docx]

**Appendix S1: Supplementary methods**

**Participants**

Children aged 0 – 16 years resident in the UK with definite or possible TSC diagnosed between 1^st^ January 2001 and 31^st^ December 2005 were ascertained through paediatricians, paediatric neurologists and clinical geneticists and the UK Tuberous Sclerosis Association by mailing at the start of the recruitment period and annually thereafter. Current diagnostic criteria were used (Roach *et al.*, 1999) and cases with a possible diagnosis of TSC were included because young children with TSC do not always meet the criteria for a definite diagnosis when they first present. Written informed consent was obtained.

In order to check for retention bias between phases, differences in demographics and risk factors were examined between participants that were assessed at Phase 1 and Phase 2 (retained, n=85, completed both Phase 1 and Phase 2; 3 participants who had not completed Phase 1 assessments were seen at Phase 2 assessments), and those who had an estimated IQ for Phase 1 but could not be contacted for assessment at Phase 2 (not retained, n=36). There was no significant difference in the distribution of mutation type (x^2^=1.16, p=.28), sex (x^2^=0.48, p=.48), history of epilepsy (x^2^=0.25, p=.62), history of epileptic spasms specifically (x^2^=1.05, p=.59), nor estimated IQ at phase 1 (F (1,119) = 0.07, p=.79). There was also no significant difference in the age at seizure onset, although a trend emerged (F (1, 104) = 3.13, p=.08; retained mean=9.80 months, SD=11.05; not retained mean= 16.69 months, SD=29.21), whereby those participants seen in both phases had a lower age at seizure onset.

**Epilepsy severity**

Of 125 participants, 114 (91%) had a history of epilepsy. A detailed seizure history was obtained from the parents using a specially devised epilepsy interview schedule that enquired about the manifestations of possible seizures (full description in Humphrey et al. 2014; see References). Parents were also given a seizure diary to record seizure type and frequency over a two-week period, as well as the drug regime and medication changes. Details from the parent interview were cross-checked against and supplemented with information from contemporaneous medical records and the summary information was used to determine the key features of the epilepsy. Medical notes and parent narratives were reviewed and scored by independent raters (Phase 1: PFB, MC, BN; Phase 2: HL, PFB, FO). Consensus coding was established by two of the raters reviewing the narratives. For most of the patients there were multiple sources of information for each time period. When there was a disagreement, additional information from doctors and the family was obtained to identify the most valid score. Epilepsy severity was assessed for the first year of life, second year of life, current Phase 1 (preceding 3 months) and current Phase 2 (preceding 3 months), with a range of 0-3 points for each feature included. Status epilepticus did not load onto the latent factor and therefore was not retained in the analysis (see Section 3 and Figure S3).

**Cortical tuber load**

Copies of clinical brain scans were obtained from the hospitals where imaging had been conducted during Phase 1, when possible (n=86). During Phase 2, more recent clinical scans were obtained for 41 participants and new scans for 23 participants were obtained (total n=109). Total tuber count ranged from 0-68 (Figure S1). Figure S2 shows results of the factor analysis of each major lobe on a latent factor for tuber burden.

**Intellectual ability and adaptive behaviour**

**Phase 1**

Intellectual abilities were assessed using the Mullen Scales of Early Learning (MSEL) in participants up to 68 months of age (n=55). The assessments were undertaken at or around the age of 2 years or at recruitment if the child was over 2 years of age at the time of diagnosis. Adaptive level was assessed using the Vineland Adaptive Behaviour Scales extended survey parental interview (n=113). Vineland adaptive composite scores were used to estimate IQ if the child was above the recommended age for administration of the Mullen Scales (n=35). In addition, the Vineland adaptive composite score was used when administration of the Mullen Scales was not possible (n=5), or when the Mullen Scales standard score was at ‘floor’ level (n=26).

**Phase 2**

The Wechsler Abbreviated Scale of Intelligence – Second Edition (WASI-2) was administered where possible (n=57). Full-scale IQ from two sub-tests (vocabulary, matrix reasoning) was used for two participants (n=2). One participant completed the British Picture Vocabulary Scale (BPVS). The Vineland Adaptive Behaviour Scales, Second Edition (VABS-II) were administered to 81 participants. The adaptive behaviour composite from the VABS-II was used to estimate IQ when the participant was not able to complete the WASI-2 or the BPVS, either due to level of functioning (n=24) or when administration of the WASI-2 was not possible (n=2). All four sub-tests of the WASI-2 were administered to 35 unaffected siblings of the TSC participants.

**Estimated IQ**

There was a moderate correlation between the WASI-2 and VABS-II scores at Phase 2 (n=54; rho=.50, p<.001), and between the MSEL at Phase 1 and the WASI-2 scores at Phase 2 (n=35, 59% of sample; rho=.69, p<.001). Estimated IQ at Phase 1 and Phase 2 were moderately correlated (n=85; rho=.60, p<.001).
