# Supplementary material for: Long‐term cognitive outcomes in tuberous sclerosis complex
Source: Dev Med Child Neurol. 2019 Sep 19;62(3):322–9. doi: 10.1111/dmcn.14356 (PMC7027810; doi:10.1111/dmcn.14356)
Supplement: Supplementary file 2 — Appendix S2: Structural equation modelling with age at seizure onset. [file DMCN-62-322-s002.docx]

**Appendix S2: Structural equation modelling with age at seizure onset**

The model yielded a good fit to the data (x^2^ (31)=45.43; p=.04; RMSEA = 0.06 (90% CI=0.01-0.10); standardized RMR= 0.04, CFI = 0.99). All significant direct paths are shown in Figure S4.

The strongest pathway (a) was indicated through type of genetic mutation, through tuber load, through non-spasm seizure severity in the first two years, to IQ at Phase 2 (β=-4.75, 95% CI -9.35 -1.69). Additional pathways were demonstrated through: (b) mutation, to tuber load, to non-spasm seizure severity, to Phase 1 IQ, through to Phase 2 IQ (β =-1.04, 95% CI -2.78 -0.34); and (c) through mutation, to tuber load, to spasm severity, to Phase 1 IQ, through to Phase 2 IQ (β =-0.58, 95% CI -2.31 -0.12).

Comparisons of these indirect effects revealed that pathway (a) through non-spasm seizure severity through to IQ at Phase 2 was significantly larger than pathway (b) through non-spasm seizure severity to IQ at Phase 1 (β =-0.90, 95% CI= -2.68 -0.25).
