# Supplementary material for: Long‐term cognitive outcomes in tuberous sclerosis complex
Source: Dev Med Child Neurol. 2019 Sep 19;62(3):322–9. doi: 10.1111/dmcn.14356 (PMC7027810; doi:10.1111/dmcn.14356)
Supplement: Supplementary file 3 — Appendix S3: Structural equation modelling for Mullen Scales of Early Learning at phase 1 and Wechsler Abbreviated Scale of Intelligence, Second Edition at phase 2. [file DMCN-62-322-s003.docx]

**Appendix S3: Structural equation modeling for Mullen Scales of Early Learning at phase 1 and Wechsler Abbreviated Scale of Intelligence (2nd edn) at phase 2**

The model yielded a good fit to the data (x^2^ (24)=37.63; p=.04; RMSEA = 0.07 (90% CI=0.02-0.11); standardized RMR= 0.08, CFI = 0.99). All significant direct paths are shown in Figure S5.

Three indirect mediation paths were significant. The strongest pathway (a) was indicated through type of genetic mutation, through tuber load, through non-spasm seizure severity in the first two years, to WASI-2 FSIQ at Phase 2 (β =-2.82, 95% CI -6.75 -0.64), explaining 31% of the total indirect effects. Additional pathways were demonstrated through: (b) mutation, to tuber load, to non-spasm seizure severity, to MSEL at Phase 1, through to WASI-2 at Phase 2 (β =-1.23, 95% CI -4.35 -0.33); (c) through mutation, to tuber load, to spasm severity to MSEL at Phase 1, through to WASI-2 Phase 2 (β =-0.82, 95% CI -2.60 -0.09).

Comparisons of these indirect effects revealed that the indirect effect (a) through non-spasm seizure severity through to IQ at Phase 2 was significantly different from the indirect effect (b) through non-spasm seizure severity to IQ at Phase 1 (β =1.36, 95% CI= 0.39 4.28) and the indirect effect (c) through epileptic spasms and IQ at Phase 1 (β=0.95, 95% CI= 0.22 2.70).
