# Supplementary material for: Long‐term cognitive outcomes in tuberous sclerosis complex
Source: Dev Med Child Neurol. 2019 Sep 19;62(3):322–9. doi: 10.1111/dmcn.14356 (PMC7027810; doi:10.1111/dmcn.14356)
Supplement: Supplementary file 4 — Appendix S4: Structural equation modelling for the Vineland Adaptive Behaviour Scale at phases 1 and 2. [file DMCN-62-322-s004.docx]

**Appendix S4: Structural equation modeling for the Vineland Adaptive Behaviour Scale at phases 1 and 2**

The model yielded a good fit to the data (x^2^ (24)=34.40; p=.07; RMSEA = 0.06 (90% CI=0.01-0.10); standardized RMR= 0.05, CFI = 0.99). All significant direct paths are shown in Figure S6.

Five indirect mediation paths were significant. The strongest pathway (a) was indicated through type of genetic mutation, through tuber load, through to VABS at Phase 2 (β =-4.27, 95% CI -11.27 -0.68), explaining 34.5% of the total indirect effects. Additional pathways were demonstrated through: (b) mutation, to tuber load, to spasm severity, through to VABS at Phase 2 (β =-1.36, 95% CI -4.12 -0.15); (c) mutation, to tuber load, to spasm severity to VABS at Phase 1, through to VABS at Phase 2 (β =-0.95, 95% CI -2.41 -0.36); and (d) from mutation, to tuber load, to non-spasm seizure severity, to VABS at Phase 1, through to VABS at Phase 2 (β =-0.92, 95% CI -2.69 -0.27). Comparisons of these indirect effects revealed no significant differences between these indirect pathways.
