# Supplementary material for: Long‐term cognitive outcomes in tuberous sclerosis complex
Source: Dev Med Child Neurol. 2019 Sep 19;62(3):322–9. doi: 10.1111/dmcn.14356 (PMC7027810; doi:10.1111/dmcn.14356)
Supplement: Supplementary file 5 — Appendix S5: Structural equation modelling for phase 1 estimated IQ only. [file DMCN-62-322-s005.docx]

**Appendix S5: Structural equation modeling for phase 1 estimated IQ only** **(to reproduce findings)**

The model yielded a good fit to the data (x^2^ (36)=847.38; p<.001; RMSEA = 0.02 (90% CI=0.01-0.08); standardized RMR= 0.05, CFI = 0.99). All significant direct paths are shown in Figure S7.

Two indirect mediation paths were significant. The strongest pathway (a) was indicated through type of genetic mutation, through tuber load, through non-spasm seizure severity in the first two years, to estimated IQ at Phase 1 (β =-2.01, 95% CI -4.71 -0.37), explaining 39% of the total indirect effects. A second pathway explaining 31% was demonstrated through (b) mutation, to tuber load, to spasm severity, to estimated IQ at Phase 1 (β =-1.80, 95% CI -3.94 -0.55). Comparisons of these indirect effects revealed no significant difference (β =-0.21, 95% CI -3.12 2.57)
