# Supplementary figures and images for: Long‐term cognitive outcomes in tuberous sclerosis complex
Source: Dev Med Child Neurol. 2019 Sep 19;62(3):322–9. doi: 10.1111/dmcn.14356 (PMC7027810; doi:10.1111/dmcn.14356)

**
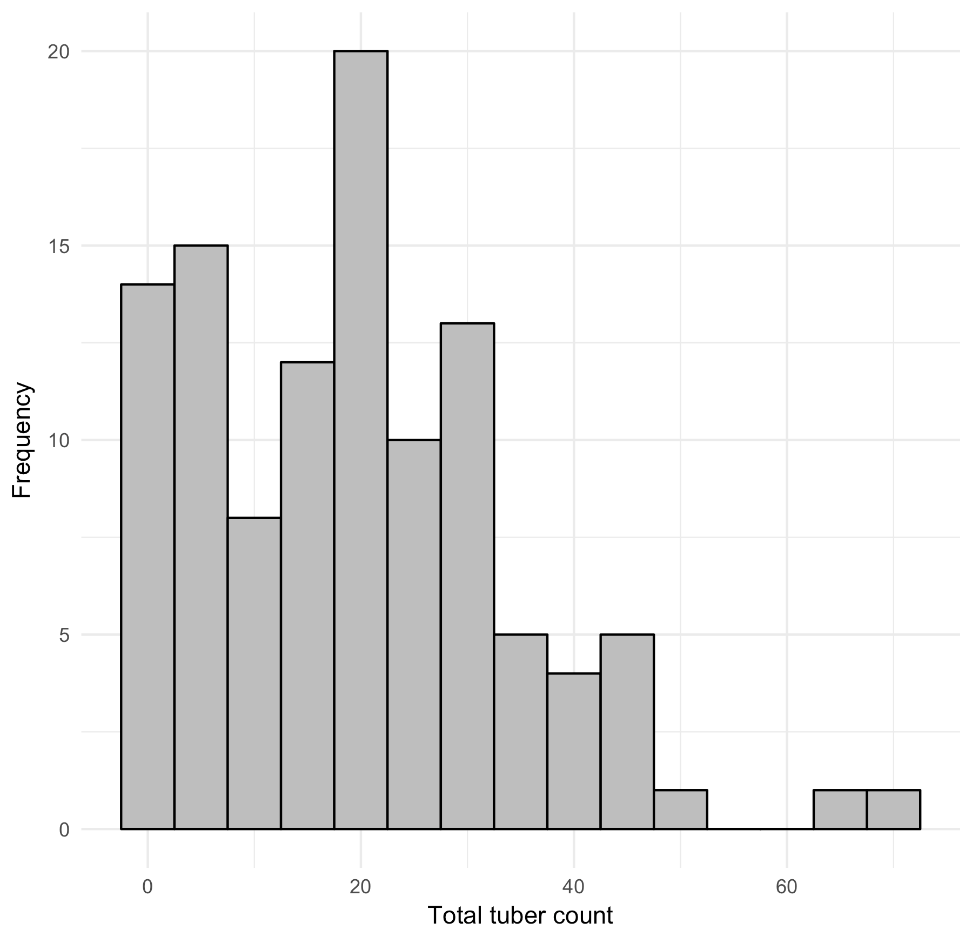
**

**Figure S1: Distribution of total tuber count across the cohort.**

Supplement: Supplementary file 6 — Figure S1: Distribution of total tuber count across the cohort. [file DMCN-62-322-s006.docx]
