# Supplementary material for: Long‐term cognitive outcomes in tuberous sclerosis complex
Source: Dev Med Child Neurol. 2019 Sep 19;62(3):322–9. doi: 10.1111/dmcn.14356 (PMC7027810; doi:10.1111/dmcn.14356)
Supplement: Supplementary file 7 — Figure S2: Factor analysis on tuber load in each lobe. [file DMCN-62-322-s007.docx]

Frontal

Temporal

Parietal

Occipital

0.94

0.75

0.84

0.71

**Figure S2: Factor analysis on tuber load in each lobe**

1-factor model: RMSEA = 0.00 (90% CI= 0.00 – 0.19); standardised RMR= 0.02; CFI = 1.00
