# Supplementary material for: Long‐term cognitive outcomes in tuberous sclerosis complex
Source: Dev Med Child Neurol. 2019 Sep 19;62(3):322–9. doi: 10.1111/dmcn.14356 (PMC7027810; doi:10.1111/dmcn.14356)
Supplement: Supplementary file 8 — Figure S3: Factor analyses on Early Childhood Epilepsy Severity Scale items at each time point. [file DMCN-62-322-s008.docx]

**Figure S3: Factor analyses on Early Childhood Epilepsy Severity Scale items at each time point**

Duration

Frequency

No. drugs

Response

0.96

0.94

0.87

0.84

Duration

Frequency

No. drugs

Response

0.89

0.94

0.83

0.86

Type

0.93

**c) Factor analysis for other seizure severity in year 1**. 1-factor model: x^2^ (5)=22.69 p<.001; RMSEA = 0.17 (90% CI=0.10-0.25); standardized RMR= 0.03, CFI = 0.97

**a) Factor analysis for epileptic spasm severity in year 1**. 1-factor model: x^2^ (2)=29.85 p<.001; RMSEA = 0.34 (90% CI=0.24-0.45); standardized RMR= 0.03, CFI = 0.94

**d) Factor analysis for other seizure severity in year 2**. 1-factor model: x^2^ (5)=9.13 p=.10; RMSEA = 0.08 (90% CI=0.00-0.17); standardized RMR= 0.02, CFI = 0.99; 2-factor model: x^2^ (1)=0.05 p=.82

**b) Factor analysis for epileptic spasm severity in year 2.** 1-factor model: RMSEA = 0.00 (90% CI=0.00-0.15); standardized RMR= 0.005, CFI = 1.00

Duration

Frequency

No. drugs

Response

0.86

0.92

0.79

0.84

Type

0.87

Response

Duration

Frequency

No. drugs

0.99

0.98

0.71

0.91

Duration

Frequency

No. drugs

Response

0.98

0.99

0.84

0.32

Type

0.98

Status epilepticus

0.94

**e) Factor analysis for seizure severity in phase 1 with status epilepticus**. 1-factor model: x^2^ (9)=60.05 p<.001; RMSEA = 0.21 (90% CI=0.16-0.26); standardized RMR= 0.11, CFI = 0.89; 2-factor model: x^2^ (4)=10.51 p=.03

Duration

Frequency

No. drugs

Response

0.98

0.99

0.83

0.94

Type

0.98

**f) Factor analysis for seizure severity in phase 1 without status epilepticus**. 1-factor model: x^2^ (5)=44.61 p<.001; RMSEA = 0.25 (90% CI=0.19-0.32); standardized RMR= 0.05, CFI = 0.91; 2-factor model: x^2^ (1)=0.33 p=.57

Duration

Frequency

No. drugs

Response

0.87

0.91

0.73

0.04

Type

0.87

Status epilepticus

0.91

**g) Factor analysis for seizure severity in phase 2 with status epilepticus**. 1-factor model: x^2^ (9)=58.14 p<.001; RMSEA = 0.24 (90% CI=0.18-0.30); standardized RMR= 0.04, CFI = 0.89. 2-factor model: x^2^ (4)=2.52 p=.64

Duration

Frequency

No. drugs

Response

0.87

0.91

0.73

0.91

Type

0.87

**h) Factor analysis for seizure severity in phase 2 without status epilepticus**. 1-factor model: x^2^ (5)=56.20 p<.001; RMSEA = 0.33 (90% CI=0.26-0.41); standardized RMR= 0.05, CFI = 0.88. 2-factor model: x^2^ (1)=0.63 p=.43

Exploratory factor analysis was conducted for each of the time periods to ascertain the loadings of each E-Chess feature of epilepsy to severity scores (Figure S3). These indicated that history of status epilepticus (none, 30 minutes to 1 hour, 1 hour or more) did not significantly load on the seizure severity factor for any time point. Status epilepticus was therefore removed from the structural equation modeling analysis. Given the reported association between intellectual development and status epilepticus, separate univariate and multivariate analyses were performed for history of status epilepticus in relation to Phase 2 estimated IQ and change in estimated IQ from Phase 1 to Phase 2, described in the main manuscript. At Phase 1, 1 participant had reported status epilepticus in previous 3 months and at Phase 2, 2 participants in previous 3 months reported status epilepticus.
