# Supplementary material for: Long‐term cognitive outcomes in tuberous sclerosis complex
Source: Dev Med Child Neurol. 2019 Sep 19;62(3):322–9. doi: 10.1111/dmcn.14356 (PMC7027810; doi:10.1111/dmcn.14356)
Supplement: Supplementary file 9 — Figure S4: Full mediation model: paths linking genotype and intellectual outcomes with age at seizure onset included. [file DMCN-62-322-s009.docx]

**FIGURE S4** (following page): Full mediation model: paths linking genotype and intellectual outcomes with age at seizure onset included. Ovals represent latent variables and rectangles represent observed variables. Absence of a line connecting variables implies no direct effect. Standardised betas for each path are shown, all paths shown are significant at p<.05.

TSC1 vs TSC2

Tuber load

Seizure severity 7y+

Estimated IQ 7y+

Estimated IQ 3y+

Seizure severity 3y+

0.22

0.31

0.39

0.36

0.35

-0.21

-0.20

-0.14

0.32

-0.36

-0.50

-0.26

0.50

0.19

Age seizure onset

-0.39

-0.19

-0.23

-0.26

Spasm y1 factor score

Spasm y2 factor score

0.50

0.90

Seizure y1 factor score

Seizure y2 factor score

0.91

0.67

0.19
