# Supplementary material for: Long‐term cognitive outcomes in tuberous sclerosis complex
Source: Dev Med Child Neurol. 2019 Sep 19;62(3):322–9. doi: 10.1111/dmcn.14356 (PMC7027810; doi:10.1111/dmcn.14356)
Supplement: Supplementary file 10 — Figure S5: Full mediation model: paths linking genotype and intellectual outcomes using Mullen Scales of Early Learning at phase 1 and Wechsler Abbreviated Scale of Intelligence, Second Edition at phase 2. [file DMCN-62-322-s010.docx]

**FIGURE S5**: Full mediation model: paths linking genotype and intellectual outcomes using Mullen Scales of Early Learning at phase 1 and Wechsler Abbreviated Scale of Intelligence (2nd edn) at phase 2, through tuber load and epilepsy severity. Ovals represent latent variables and rectangles represent observed variables. Absence of a line connecting variables implies no direct effect. Standardised betas for each path are shown, all paths shown are significant at p<.05.

TSC1 vs TSC2

Tuber load

Seizure severity 7y+

WASI-2 7y+

MSEL 3y+

Seizure severity 3y+

0.34

0.40

0.53

0.36

-0.22

-0.25

0.33

-0.37

-0.45

-0.26

0.44

0.19

Spasm y1 factor score

Spasm y2 factor score

Seizure y1 factor score

Seizure y2 factor score

0.93

0.63

0.50

0.99

0.19
