# Supplementary material for: Long‐term cognitive outcomes in tuberous sclerosis complex
Source: Dev Med Child Neurol. 2019 Sep 19;62(3):322–9. doi: 10.1111/dmcn.14356 (PMC7027810; doi:10.1111/dmcn.14356)
Supplement: Supplementary file 11 — Figure S6: Full mediation model: paths linking genotype and intellectual outcomes using the Vineland Adaptive Behaviour Scale at phases 1 and 2. [file DMCN-62-322-s011.docx]

**FIGURE S6**: Full mediation model: paths linking genotype and intellectual outcomes using the Vineland Adaptive Behaviour Scale at phases 1 and 2, through tuber load and epilepsy severity. Ovals represent latent variables and rectangles represent observed variables. Absence of a line connecting variables implies no direct effect. Standardised betas for each path are shown, all paths shown are significant at p<.05.

TSC1 vs TSC2

Tuber load

Phase 2 seizure severity

Phase 2

VABS

Phase 1

VABS

Phase 1 seizure severity

0.29

0.37

0.53

0.34

-0.30

0.31

-0.32

-0.45

-0.26

0.41

0.19

Spasm y1 factor score

Spasm y2 factor score

Seizure y1 factor score

Seizure y2 factor score

0.93

0.66

0.50

0.90

-0.19

0.18

-0.41

-0.21
