# Supplementary material for: Long‐term cognitive outcomes in tuberous sclerosis complex
Source: Dev Med Child Neurol. 2019 Sep 19;62(3):322–9. doi: 10.1111/dmcn.14356 (PMC7027810; doi:10.1111/dmcn.14356)
Supplement: Supplementary file 12 — Figure S7: Full mediation model: paths linking genotype and intellectual ability at phase 1 only. [file DMCN-62-322-s012.docx]

**FIGURE S7**: Full mediation model: paths linking genotype and intellectual ability at phase 1 only (for reproduction purposes in the smaller sample), through tuber load and epilepsy severity. Ovals represent latent variables and rectangles represent observed variables. Absence of a line connecting variables implies no direct effect. Standardised betas for each path are shown, all paths shown are significant at p<.05.

TSC1 vs TSC2

Tuber load

Estimated IQ 3y+

Seizure severity 3y+

0.32

0.38

0.40

-0.23

0.32

-0.33

0.18

Spasm y1 factor score

Spasm y2 factor score

Seizure y1 factor score

Seizure y2 factor score

0.98

0.54

0.50

0.90

0.18
