# Supplementary material for: Long‐term cognitive outcomes in tuberous sclerosis complex
Source: Dev Med Child Neurol. 2019 Sep 19;62(3):322–9. doi: 10.1111/dmcn.14356 (PMC7027810; doi:10.1111/dmcn.14356)
Supplement: Supplementary file 13 — Table S1: Clinical characteristics of sample subgroups with: (1) no change, (2) a relative increase, or (3) a relative decrease in IQ from phase 1 to phase 2 [file DMCN-62-322-s013.docx]

**Table S1**: Clinical characteristics of sample subgroups with (1) no change, (2) a relative increase, or (3) a relative decrease in IQ from phase 1 to phase 2. Raw scores are presented. Analyses are conducted on factor scores.

|  |  | **No change (n=50)** | **Faster gain in skills (n=19)** | **Slower gain in skills (n=16)** | **Group difference (slower vs faster gain in skills)** |
| --- | --- | --- | --- | --- | --- |
| **Characteristics** | Gender, n male (%) | 22 (44%) | 9 (47%) | 8 (50%) | x^2^=.02, p=.88 |
|  | ID status at Phase 1, n (%) | 31 (62%) | 14 (74%) | 11 (69%) | x^2^=.10, p=.75 |
|  | Mean estimated IQ at Phase 1 | 68.64 (20.40) | 59.05 (18.57) | 71.87 (25.73) | F=2.92, p=.10 |
|  | ID status at Phase 2, n (%) | 32 (64%) | 2 (11%) | 14 (88%) | x^2^=.20.74, p<.001 |
|  | Mean estimated IQ at Phase 2 | 67.18 (22.36) | 87.21 (13.53) | 47.13 (21.05) | F=46.32, p<.001 |
|  | Mean change in estimated IQ (SD) | 1.46 (8.10) | +24.75 (8.95) | -28.16 (6.62) | F=0.02, p=.89 |
|  | Mean interval between assessments, years (SD) | 8.23 (1.50) | 7.94 (1.58) | 9.46 (0.57) | F=11.67, p=.002 |
| **Mutation analysis** | n TSC2 (versus TSC1) (%) | 30 (73%) | 14 (86%) | 11 (90%) | x^2^=.16, p=.69 |
| **Epilepsy variables** | History of epilepsy ever, n (%) | 47 (94%) | 16 (84%) | 16 (100%) | x^2^=2.76, p=.09 |
|  | Age at seizure onset in months, median (range) | 6 (0-64) | 7 (0.75-37) | 3 (0.25-27) | U=172.50, z=2.08, p=.04 |
|  | Age at seizure onset after 12 months, n (%) | 14 (30%) | 4 (25%) | 2 (13%) | x^2^=0.82, p=.37 |
|  | History of status epilepticus in year 1 or 2, n (%) | 14 (28%) | 3 (16%) | 6 (38%) | x^2^=2.14, p=.14 |
|  | History of status epilepticus ever, n (%) | 21 (42%) | 4 (21%) | 9 (56%) | x^2^=4.61, p=.03 |
|  | History of epileptic spasms, n (%) | 30 (60%) | 6 (32%) | 9 (56%) | x^2^=.33, p=.20 |
|  | Severity of epileptic spasms in year 1, median (range) | 0 (0-12) | 0 (0-12) | 3 (0-12) | U=121.50, z=-1.12, p=.32 |
|  | Severity of epileptic spasms in year 2, median (range) | 0 (0-11) | 0 (0-11) | 0 (0-13) | U=95.50, z=-2.12, p=.06 |
|  | Severity of non-spasm seizures in year 1, median (range) | 0 (0-15) | 0 (0-10) | 9 (0-14) | U=60.00, z=-3.28, p=.002 |
|  | Severity of non-spasm seizures in year 2, median (range) | 0 (0-14) | 4 (0-13) | 11 (0-14) | U=80.50, z=-2.42, p=.02 |
|  | Seizure severity Phase 1, median (range) | 9 (0-17) | 11.5 (0-19) | 13 (0-19) | U=98.00, z=-1.80, p=.07 |
|  | Seizure severity Phase 2 , median (range) | 6 (0-14) | 3 (0-11) | 9.5 (0-15) | U=93.00, z=-2.31, p=.02 |
| **Brain involvement** | Cortical tuber load, median (range) | 18 (0-46) | 9 (0-47) | 28 (0-41) | U= 93.00, z=1.52, p=.14 |
