# Supplementary material for: Long‐term cognitive outcomes in tuberous sclerosis complex
Source: Dev Med Child Neurol. 2019 Sep 19;62(3):322–9. doi: 10.1111/dmcn.14356 (PMC7027810; doi:10.1111/dmcn.14356)
Supplement: Supplementary file 14 — Table S2: Spearman correlations between main phenotypic features [file DMCN-62-322-s014.docx]

**Table S2: Spearman correlations (rho) between main phenotypic features. ***p<.001 **p<.01 *p<.05 ^+^p<.10**

|  | **Age at seizure onset** | **Spasm severity year 1** | **Spasm severity year 2** | **Other seizure severity y1** | **Other seizure severity y2** | **Seizure severity Phase 1** | **Seizure severity Phase 2** | **Total tuber**  **count** | **WASI-2 FSIQ Phase 2** | **Vineland**  **Composite**  **Score Ph2** |
| --- | --- | --- | --- | --- | --- | --- | --- | --- | --- | --- |
| **Spasm severity y1** | -.45*** |  |  |  |  |  |  |  |  |  |
| **Spasm severity y2** | -.36*** | .75*** |  |  |  |  |  |  |  |  |
| **Other seizure severity y1** | -.67*** | .17**^+^** | .13 |  |  |  |  |  |  |  |
| **Other seizure severity y2** | -.46*** | .28** | .33*** | .61*** |  |  |  |  |  |  |
| **Seizure severity Phase 1** | -.36*** | .43*** | .37*** | .35*** | .52*** |  |  |  |  |  |
| **Seizure severity Phase 2** | -.12 | .06 | .06 | .28** | .47*** | .57*** |  |  |  |  |
| **Total**  **tuber count** | -.33** | .39*** | .30** | .19**^+^** | .31** | .18**^+^** | .01 |  |  |  |
| **WASI-2 FSIQ Phase 2** | .26**^+^** | -.23**^+^** | -.35** | -.32* | -.57*** | -.45*** | -.30* | -.18 |  |  |
| **VABS-II**  **Composite**  **Score Phase 2** | .23**^+^** | -.35** | -.35** | -.31** | -.46*** | -.58*** | -.54*** | -.15 | .50*** |  |
| **Estimated**  **IQ** | .38** | -.33** | -.43** | -.49*** | -.63*** | -.54*** | -.49*** | -.24* | 1.00*****⌃** | .80*** |

**Key**: **^+^**p<.10; *=p<.05; **p<.01; ***p<.001. ⌃=Estimated IQ is equal to WASI-2 FSIQ in individuals who completed the WASI-2
