# Supplementary material for: Long‐term cognitive outcomes in tuberous sclerosis complex
Source: Dev Med Child Neurol. 2019 Sep 19;62(3):322–9. doi: 10.1111/dmcn.14356 (PMC7027810; doi:10.1111/dmcn.14356)
Supplement: Supplementary file 15 — Table S3: Group differences by genotype [file DMCN-62-322-s015.docx]

**Table S3: Group differences by genotype (median, range or mean, SD of raw scores, group differences based on factor scores)**

|  | **TSC1 (n=19)** | **TSC2 (n=77)** | **Group difference** |
| --- | --- | --- | --- |
| Age at seizure onset (months) | 20 (3-122) | 5 (0-60) | **U = 246.50, z=-3.40 p=.001** |
| Spasm severity year 1 | 0 (0-9) | 0 (0-12) | **U = 967.00, z=2.85, p=.004** |
| Spasm severity year 2 | 0 (0-9) | 0 (0-12) | **U = 902.50, z=2.10, p=.036** |
| Other seizure severity year 1 | 0 (0-11) | 0 (0-15) | U = 798.00, z=1.44, p=.151 |
| Other seizure severity year 2 | 0 (0-12) | 6 (0-14) | **U = 863.00, z=1.98, p=.048** |
| Seizure severity Phase 1 | 2 (0-17) | 10 (0-19) | U = 895.00, z=1.52, p=.128 |
| Seizure severity Phase 2 | 9.50 (0-15) | 6 (0-14) | U = 337.00, z=-1.16, p=.246 |
| Total tuber count | 4 (0-22) | 22 (0-68) | **U = 844.00, z=3.95, p<.001** |
| WASI-2 FSIQ Phase 2**⌃** | 92.40 (19.06) | 74.73 (17.78) | **F (1,48) = 7.69, p=.008** |
| Vineland Composite Score Phase 2 | 65.50 (17.47) | 66.60 (20.92) | F (1,60) = 0.03, p=.86 |
| Estimated IQ | 76.44 (26.81) | 67.25 (21.20) | F (1,67)=2.04, p=.16 |

⌃: WASI-2 FSIQ only available in able subset of individuals (n=50 with mutation data)
